# Laccase-Mediated Incorporation of Xylans and Lignin-Carbohydrate Complexes into High-Yield Eucalyptus Kraft Fibers

**DOI:** 10.1021/acsomega.5c00812

**Published:** 2025-04-17

**Authors:** Uirajá Cayowa Magalhães Ruschoni, Pedro Jorge Fonseca Chagas, Pieter De Wever, Samuel Eyley, Wim Thielemans, Adriane Maria Ferreira Milagres, Pedro Fardim, André Ferraz

**Affiliations:** † Departamento de Biotecnologia, Escola de Engenharia de Lorena, Universidade de São Paulo, 12602-810 Lorena, SP, Brazil; ‡ Department of Chemical Engineering, KU Leuven, Celestijnenlaan 200F, 3001 Leuven, Belgium; § Sustainable Materials Laboratory, Department of Chemical Engineering, KU Leuven, Kulak Kortrijk Campus, Etienne Sabbelaan 53, 8500 Kortrijk, Belgium

## Abstract

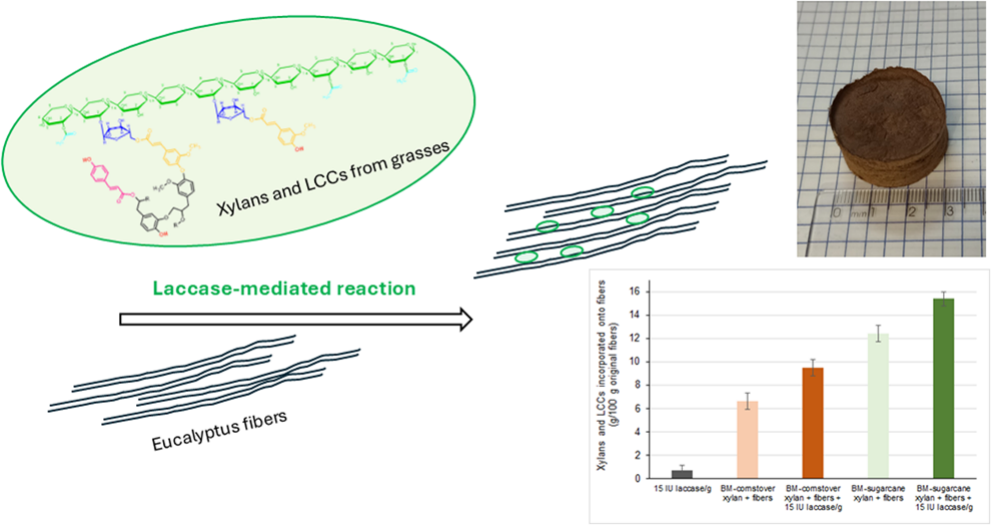

Water-soluble products derived from biomass processing hold significant potential to enhance biorefinery viability due to their versatile applications. However, their use in the modification of paper products remains underexplored. Here, corn stover and sugarcane bagasse provided water-soluble products being incorporated into high-yield eucalyptus kraft pulps. The water-soluble products were extracted from ball-milled or alkaline-sulfite pretreated biomass using a xylanase-assisted procedure. Feruloylated arabinoxylans and lignin-carbohydrate complexes (LCCs) predominated in the extracted mixtures. The water-soluble products were incorporated into the fibers using laccase-mediated reactions. The fibers incorporated up to 15.4 g of water-soluble products/100 g of pulp, depending on the biomass source and pretreatment type. The highest incorporation levels were achieved with the water-soluble products recovered from ball-milled sugarcane bagasse. This fraction underwent significant polymerization of feruloylated arabinoxylans during laccase treatment. Paper sheets prepared from fibers with high levels of xylan and LCCs exhibited decreased water contact angles, indicating their potential suitability for a more wettable brown tissue paper. High-resolution X-ray photoelectron spectroscopy revealed an increase in the content of carboxyl and ester groups, corroborating the incorporation of xylans and LCCs into the fibers. The study presents novel strategies for modifying fiber properties through the use of agricultural byproducts.

## Introduction

Oligosaccharides, hemicelluloses, and lignin-carbohydrate complexes (LCCs) are among the water-soluble products derived from biomass processing that remain underexplored for enhancing biorefinery performance.^1−3^ These products usually comprise a complex mixture of molecules that could barely be purified before their final use. Complex mixtures of biomass fractions lack the advantages of pure products but, in contrast, open the opportunity for developing new materials with low production costs.^2^ Direct use of these biomass-derived mixtures as antioxidants, antimicrobial agents, biocarriers, bioplastics, and composites has been reported.^4−7^

Potential applications for complex mixtures of biomass fractions include incorporation in pulp slurries or paper surfaces to increase product yield or develop innovative biomass-derived materials with enhanced demand. Examples of innovative materials include cellulose-based packing products designed to replace plastics,^8,9^ cellulose-based composites for insulating materials,^10^ and new paper products with distinguished characteristics.^11−13^ In the last subject, brown tissue paper has emerged as a new product with ecological appeal because it includes unbleached virgin fibers or recycled fibers from corrugated containerboard.^12,14,15^ However, bleached fibers are still preferred as the major pulp for tissue paper production considering their outstanding consumer acceptance, high wettability, and high strength.^12^ Unbleached fibers or high-lignin content fibers tend to absorb less water and to produce weaker paper owing to the occurrence of hydrophobic lignin covering the fiber surfaces.^12,16^ Incorporation of hemicelluloses and LCCs into these fibers is then a logical choice because these biorefinery-derived products are hydrophilic owing to their high carbohydrate contents.^1,5,17^ In addition to the polysaccharide backbone, hemicelluloses and LCCs may contain phenolic side groups, such as ferulate in grass-derived hemicelluloses and lignin moieties in LCCs.^1,2,5^ LCCs and hemicelluloses derived from grass lignocellulosic materials can also help enhancing the strength of paper produced from lignin-containing fibers because these components afford phenolic hydroxyl, which are suitable for oxidation by laccases, producing phenoxyl radicals.^18,19^ Multiple cross-links of these phenoxyl radicals with lignin present on the fiber surfaces could enhance paper strength associated with stronger fiber–fiber interactions as already demonstrated for laccase-treated thermomechanical pulps used to produce medium-density fiberboards.^20^

In the current work, two relevant industrial crop byproducts, namely, sugarcane bagasse and corn stover, supplied water-soluble products within a biorefinery context. The pretreated biomass materials were extracted in a xylanase-assisted step to yield a mixture of water-soluble xylans and LCCs. Xylanase-assisted extraction was employed due to its mild reaction conditions, which prevent the degradation of ferulate side chains in hemicelluloses and lignin-carbohydrate linkages in LCCs. Extracted xylans and LCCs were then incorporated into unbleached high-yield eucalyptus kraft pulp fibers with the aid of a laccase-mediated process. In summary, this work demonstrates the valorization of biorefinery byproducts by supporting the use of biomass-derived water-soluble fractions to enhance the yield and quality of pulp slurries, which are useful to produce brown tissue paper.

## Materials and Methods

### Plant Biomass Resources

Sugarcane bagasse was provided by a local sugarcane mill located at Quatá city, SP, Brazil (22°14′51″ S, 50°41′54″ W). Corn stover (plant stems plus leaves left in the field after maize harvesting) is derived from a commercial maize variety, named Agr1051. Plant stems were hand-cut directly from a plantation field located at Canas city, SP, Brazil (22°42′13″ S, 45°03′19″ W). Wet fibrous materials were air-dried to a humidity level of 10–12%, milled using a coarse knife mill, and stored under dry conditions until use in the described experiments. *Eucalyptus urograndis* wood chips were provided by a local pulp mill located at Jacareí city, SP, Brazil (23°18′10″ S, 45°17′31″ W). Xylan from oat spelt used as a reference material was purchased from Fluka-BioChemica (#95590).

### Pretreatment of Sugarcane Bagasse and Corn Stover

For the ball-milling (BM) pretreatment, crude sugarcane bagasse and corn stover were previously cut in a knife mill to pass a 20-mesh screen (Manesco & Ranieri, Brazil). This milled material was extracted with 95% (v/v) ethanol in a Soxhlet apparatus for 8 h. The extractive-free material was air-dried and milled to flour appearance in a 3.7 L stainless steel jar set in the ball-milling equipment (Quimis Q298, Brazil). Each 90 g of extractive-free material was crushed by 1.8 kg of 18 mm stainless balls for 130 h at 98 rpm.

For the alkaline-sulfite chemithermomechanical pretreatment (AS-CTMP), the procedures described in a previous paper were used.^21^ In summary, 1.2 kg of crude plant biomass materials were treated with 7.5% Na_2_SO_3_ and 3.75% NaOH (w/w on plant biomass, oven dry basis) in a solid-liquor ratio of 1:10 (w/v) at 127 °C for 2 h, followed by disk refining set to a net energy consumption of 650 kWh (MD-300 Regmed, Brazil). Pretreated materials were used in the subsequent end-oxylanase-assisted extraction experiments.

### Endoxylanase-Assisted Extraction of the Water-Soluble Fractions

Endoxylanase-assisted extraction procedures employed 90 g of pretreated solids suspended in water at 50 g/L. The suspension pH was set to 8.0 with 1 mol/L NaOH and a commercial endoxylanase (Luminase PB-200, BASF, Brazil) was added (8 IU/g substrate). After shaking at 120 rpm and 50 °C for 24 h, the mixture was treated in a boiling water bath for 5 min for enzyme denaturation.^1^ The suspension was filtered through porous glass filter no. 2 and then 0.45 μm membranes. The pH of the water-soluble fractions was adjusted to 7 before freeze-drying. The resulting crude freeze-dried material was weighed and stored at room temperature for use in subsequent experiments.

### Preparation of High-Yield Eucalyptus Kraft Pulp (HYEKP) and Bleached Eucalyptus Pulp Fibers Resource

*E. urograndis* wood chips were cooked with a mixture of NaOH and Na_2_S, with 13% active alkali (w/w, based on oven-dry wood) and 25% sulfidity. The cooking was performed inside a 20 L rotating reactor (AU/E-20 - Regmed, Brazil) operated with a wood- liquor ratio of 1:4 (w/v).^22^ The maximal cooking temperature of 170 °C was attained after a 50 min heating period and maintained at 170 °C for an additional 3 h, followed by reactor cooling down to 90 °C before opening. Cooked fibers were recovered by centrifugation through a nylon screen and defibrated at 75 g/L for 30 s in a blender. Defibrated solids were further refined in a disk refiner at 18 g/L using 0.1 mm disk clearance up to a net energy consumption of 500 kWh (MD-3000 – Regmed, Brazil). The refined pulp was screened in a water suspension using a 0.15 mm slot screen. The recovered fibers (HYEKP) were centrifuged through a nylon screen and stored at 4 °C for subsequent experiments.

Fully bleached eucalyptus pulp fibers (90% ISO brightness) used as a reference material were kindly provided by a local pulp mill processing *E. urograndis*.

### Treatment of Water-Soluble Fractions Recovered from Pretreated Sugarcane Bagasse and Corn Stover with Laccase from *Trametes versicolor*

The laccase from *T. versicolor* originates from a previous work.^23^ The enzyme activity of the concentrated preparation was periodically assayed using 2,2′-azino-bis(3-ethylbenzothiazoline-6-sulfonic acid) as substrate following standard procedures.^24^ The average activity of the currently used laccase was 430 IU/mL. The concentrated extract also contained low hydrolytic activities of endoglucanases (0.12 IU/mL), β-glucosidase (1.7 IU/L), and β-xylosidase (1.1 IU/L). Endoxylanase was assayed in the extract but was not detectable.^23^

For the laccase-mediated reactions, 20 mg of each freeze-dried extract obtained by the endoxylanase-assisted extraction procedure was dissolved in 12 mL of water, and the solution pH was adjusted to 5.0 with 1 mol/L H_2_SO_4_. The solutions were transferred to 25 mL Erlenmeyer flasks, and *T. versicolor* laccase was added to a proportion of 200 IU/g substrate. The reactions were carried out at 30 °C for 2 h at 120 rpm in 25 mL Erlenmeyer flasks with loosely capped lids, in a rotary shaker. After the reaction completed, a nitrogen stream was fluxed into the flasks to remove dissolved O_2_, stopping laccase action. Reaction products were stored at −18 °C before characterization.

### Chemical and Spectroscopic Characterization of the Water-Soluble Fractions Recovered from Pretreated Sugarcane Bagasse and Corn Stover

Freeze-dried solids were characterized to determine polysaccharide composition, lignin content, and *p*-hydroxycinnamate contents following previously described protocols.^25,26^ Severe alkaline reactions released the total *p-hydroxycinnamates*, whereas mild reactions released only the ester-linked *p*-hydroxycinnamates containing phenolic-free hydroxyls.^19^

UV/vis spectra were recorded in an Evolution 201 spectrophotometer (Thermo Scientific) from samples dissolved in water at final concentrations of 0.2–0.5 mg/mL. Differential UV/vis spectra were calculated from the same samples dissolved in aqueous solutions at pHs 13.0 or 1.0, and used to determine total phenolic group contents.^27^

Size exclusion chromatography was performed with 1 mg/mL samples in a Superose 12 10/300 column (GE HealthCare). 250 μL of each sample were eluted in 10 mM NaOH at 0.6 mL/min. The eluted material was detected online at 280 nm (Akta Purifier, GE HealthCare). One milliliter samples eluting from the column were collected and assayed for carbohydrates using the phenol-sulfuric acid reagent, with detection at 490 nm.^1^ The column system was calibrated using raffinose (504 g/mol), dextrans from 1000 to 10,000 g/mol, and proteins from 12,384 to 66,000 g/mol, all from Sigma-Aldrich (Merck-Sigma-Aldrich).

### Incorporation of the Water-Soluble Fractions Recovered from Pretreated Sugarcane Bagasse and Corn Stover into HYEKP Fibers via Laccase-Mediated Reactions

HYEKP fibers (2 g, oven-dry basis) were suspended in 100 mL of 100 mM sodium acetate buffer at pH 5.0 inside 250 mL Erlenmeyer flasks with internal baffles and loosely capped lids. To this suspension, 0.3 g of the freeze-dried products extracted through the endoxylanase-assisted procedure were added. Laccase (15 IU/g of fibers) was added to the mixture and incubated at 40 °C and 200 rpm for 4 h in a rotary shaker. After treatment, the fiber suspension was vacuum filtered through sintered glass filters (Schott #3) and washed with 200 mL of water. After washing, the fibers were pressed with a 1 kg stainless steel solid billet under vacuum for 1 min (Figure S1). Pressed fibers were dried at 60 °C for 16 h and then at 100 °C for an additional 4 h and then weighed. A set of samples was also prepared using a similar procedure but without the addition of laccase, serving to assess the adsorption of the freeze-dried products onto the fibers.

After weight determination, the test specimens were also used to estimate the strength of the fiber–fiber interactions. For this assessment, each pressed and dried fiber mat was allowed to swell by immersion in water for 4 h. Swollen test specimens were disintegrated in a pulp blender for 3.3 min (10,000 revolutions) following TAPPI standard 205. Disintegrated pulp suspension was classified in a 0.2 mm screen for 4 min. Fiber shives retained in the screen were recovered and dried at 105 °C up to a constant weight. The mass ratio between fiber shives and original pulp provided the fraction of fiber shives resisting disintegration. A reference assay used 0.3 g of commercial poly(vinyl alcohol) glue (3M, Brazil) to indicate maximal levels of shives formation under this experimental protocol.

### Preparation of Paper Test Specimens with HYEKP Fibers and Water-Soluble Fractions Recovered from Pretreated Sugarcane Bagasse and Corn Stover in Laccase-Mediated Reactions

HYEKP fibers were suspended in sodium acetate buffer (100 mM, pH 5.0) at 30 g/L and disintegrated for 5 min (15,000 revolutions) in a pulp blender following TAPPI standard 218-sp02. Prepared pulp suspension was maintained under magnetic stirring, and a 57 mL sample (175 mg of fibers on a dry basis) was mixed with 27.5 mg of water-soluble products provided through the endoxylanase-assisted extraction procedure. Laccase was then added to the mixture. All reactions were carried out at 40 °C for 4 h under 200 rpm in a rotary shaker. The mixture was then filtered through filter paper held in Millipore filtration glassware (Merck Millipore), producing a paper sheet test specimen with 175 g/m^2^. The resulting paper sheet was pressed twice at 2.03 kN for 5 min, dried at 25 °C for 48 h (TAPPI T218-sp02), and then wrapped up with aluminum foil. Two control paper sheet samples were prepared as follows: one with fibers and laccase in absence of the water-soluble products provided through the endoxylanase-assisted extraction procedure and another with fibers plus the water-soluble products which were provided through the endoxylanase-assisted extraction procedure without laccase addition.

### X-ray Photoelectron Spectroscopy Characterization and Apparent Water Contact Angle Determination of the Paper Test Specimens

X-ray photoelectron spectroscopy (XPS) of acetone-extracted samples was performed on a Kratos Axis Supra photoelectron spectrometer with a monochromated Al Kα (1486.69 eV, 120 W) X-ray source, hybrid (magnetic/electrostatic) optics, and a hemisphere analyzer. The analyzer was operated in fixed analyzer transmission mode with survey spectra taken at a pass energy of 160 eV and high-resolution spectra at a pass energy of 20 eV. All spectra were acquired at normal emission under charge-neutralizing conditions using a low-energy electron gun within the field of the magnetic lens. Three areas per sample were measured, and the results are reported as a mean and standard deviation of those measurements.

Spectra were subsequently processed using CasaXPS. The binding energy scale was referenced to aliphatic carbon (C–C) at 285.0 eV, and high-resolution spectra were fitted with peak models consisting of groups of symmetric component peaks.

Quantification was performed using relative sensitivity factors based on Scofield photoelectron cross sections and corrected for the electron attenuation length using the universal curve by Seah.^28^ The spectrometer transmission was corrected for using an NPL transmission function.^29^ The resulting elemental concentrations do not consider surface nanostructure and should be considered as a homogeneous equivalent composition.

An acetone-extracted filter paper sample (Whatman no. 4, #1004090, Cytiva, China) was used in a reference experiment. The O/C ratio detected in this sample was 0.75 ± 0.01. The O/C ratios for lignin (0.33) and polysaccharides (0.83) were used for theoretical O/C ratios calculations.^30^

The C_1s_ spectra provided partial curves of alkyl carbon (C_1_: C–C or C–H), carbons with one bond with oxygen (C_2_: C–O), carbons with two bonds with oxygen (C_3_: O–C–O or C=O), and carbons with three bonds with oxygen (C_4_: O–C=O). The reference of binding energy for C_1s_ was 285.0 ± 0.0 eV. Deconvolution of the other peaks takes the shift of +1.8 ± 0.0 eV for C_2_, + 3.1 ± 0.0 eV for C_3_, and +4.0 ± 0.2 eV for C_4_ from C1. Lignin-containing samples showed a fifth component peak due to π*←π shakeup transitions in aromatic species (C_5_).

The water contact angle with the paper surfaces was measured using a Theta Flex Optical Tensiometer (Biolin Scientific, Sweden) equipped with precision polypropylene needles (length 12.7 mm, diameter 0.48 mm, #7018205, Nordson EDF). Data acquisition was performed with the software OneAttention supplied with the equipment. Measurements were taken using the sessile drop mode, with the analysis set to evaluate contact angle based on the Young–Laplace method.^31^ Frames were recorded at a rate of 6.9 FPS for 5 min. For each sample, three measurements were conducted, and the baseline was determined manually. The apparent contact angle was calculated from each experimental data set relating the contact angle with time, extrapolating the linear region measured between 0.5 and 4 s to the zero time.^32^

## Results and Discussion

### Xylanase-Assisted Extraction of Xylans and Lignin-Carbohydrate Complexes (LCCs) from Pretreated Lignocellulosic Materials and Their Cross-Linking Reactions Mediated by Laccases

Two representative agricultural byproducts, sugarcane bagasse and corn stover, were initially pretreated using ball-milling (BM) or alkaline-sulfite chemithermomechanical processes (AS-CTMP) to produce digestible substrates suitable for hemicellulose extraction in aqueous media, assisted by alkali-active endoxylanases.^1^ BM provides pretreated materials with a flour appearance and high surface area, preserving most of the original lignocellulose chemical structures.^33^ The particle size distribution of the ball-milled sugarcane bagasse and corn stover showed that over 90% of the particles were less than 3.38 and 2.57 μm, respectively (Figure S2). Their surface areas were, respectively, 235 and 517 m^2^/kg. AS-CTMP, using relatively low chemical loads (7.5% w/w Na_2_SO_3_ and 3.75% w/w NaOH),^21^ produced pretreated substrates that were partially delignified and contained a residual xylan depleted in acetyl groups (Table S1).

Digestion of the pretreated materials with a low dosage of alkali-active endoxylanase (8 IU/g substrate) yielded water-soluble products whose UV/vis spectra showed prominent bands at 280 and 315 nm, corresponding to lignin or lignin-carbohydrate complexes (LCCs) and *p*-hydroxycinnamate structures, respectively (Figure S3).^34^ A commercial oat spelt xylan, exhibiting very low absorptivity in the UV region, was used in subsequent experiments as a lignin-free reference sample. The absorptivity values at 280 nm for the prepared samples ranged from 2.9 to 5.0 L/g·cm, which are lower than the typical lignin absorptivity values of 15–28 L/g·cm,^21,35^ suggesting that aromatic components were not the major fractions in the evaluated samples.

The chemical composition of the crude freeze-dried extracts confirmed that the endoxylanases primarily extracted xylans while also solubilizing LCCs, water-soluble lignin fractions, ash, and glucan-containing oligosaccharides in varying amounts (Table 1). Ash in freeze-dried samples originates from plant ashes solubilized in the extraction procedure plus residual salts from pretreatment and/or pH adjustment steps used during extraction. Glucan can originate from starch, mixed-linkage glucans, and other soluble glucan oligosaccharides present in the original grass samples.^36,37^*p*-Coumarate was detected in all samples and is associated with the lignin fraction of maize and sugarcane secondary cell walls, while the detected ferulates are typically esterified to the arabinosyl side groups of xylan structures.^38^

**Table 1:** Chemical Composition of Soluble Fractions Released by Alkaline-Active Endoxylanases^a^ from Ball-Milled (BM) and Alkaline-Sulfite Chemithermomechanically Pretreated (AS-CTMP) Sugarcane Bagasse and Corn Stover

	structural xylan components (g/100 of solids after lyophilization)					lignin fraction and nonstructural xylan components (g/100 of solids after lyophilization)				
biomass resource/pretreatment	anhydrous xylose	arabinosyl	acetyl	etherified ferulate	phenolic-free ferulate	total lignin	etherified *p*-coumarate	phenolic-free *p*-coumarate	gluca	ash
Sugarcane Bagasse										
BM	42.6 ± 0.3	5.1 ± 0.1	5.8 ± 0.6	0.51 ± 0.04	1.29 ± 0.05	10.4 ± 0.1	0.37 ± 0.03	0.69 ± 0.02	10.1 ± 0.1	5.9
AS-CTMP	52.8 ± 0.3	7.0 ± 0.3	0.51 ± 0.1	0.85 ± 0.02	0.11 ± 0.01	17.7 ± 0.1	2.93 ± 0.07	0.91 ± 0.04	2.3 ± 0.2	5.4
Corn Stover										
BM	26.8 ± 0.1	4.5 ± 0.5	2.6 ± 0.5	0.67 ± 0.09	0.70 ± 0.06	18.0 ± 0.1	0.26 ± 0.09	0.15 ± 0.04	14.9 ± 0.4	18.0
AS-CTMP	34.6 ± 1.4	6.3 ± 0.2	1.0 ± 0.3	1.00 ± 0.09	0.06 ± 0.02	20.6 ± 0.1	1.6 ± 0.09	nd	2.7 ± 0.1	11.2

a Crude extraction yields corresponded to 4.5–8 g of solubilized material/100 g of pretreated plant biomass. nd: not detected, below detection limit of the current methodology.

Xylose, arabinose, acetic acid, and ferulic acid were major building blocks in the hemicellulose backbones (Table 1). Molar ratios of these constituents indicated a predominance of arabinosylated xylan structures (Table 2), which are typical of grass secondary cell walls.^38^

**Table 2:** Molar Ratios of Xylan Constituents Based on One Hundred Anhydrous-Xylose Chain^a^

	molar proportion of constituent monomers				
xylan source/pretreatment	xylose	arabinose	acetic acid	etherified ferulic acid	phenolic-free ferulic acid
Sugarcane Bagasse					
BM	100	12	42	1	2
AS-CTMP	100	13	3	1	0.1
Corn Stover					
BM	100	17	30	2	2
AS-CTMP	100	18	9	1.8	0.1

a Xylans were released by alkaline-active endoxylanases from ball-milled (BM) and alkaline-sulfite chemithermomechanically pretreated (AS-CTMP) sugarcane bagasse and corn stover.

4-*O*-Methyl glucuronic acid is also a common side group in sugarcane and maize xylans,^1,38^ but it was not analyzed in the current samples. Acetyl groups were preserved in the samples extracted from BM substrates but not in those recovered from AS-CTMP substrates, as the alkaline conditions in the AS-CTMP step saponified the esters.^39^ Accordingly, ferulate containing phenolic-free groups was significant only in extracts recovered from BM substrates (2 PhOH-FA/100 Xyl in both samples), in contrast with 0.1 PhOH-FA/100 Xyl in the xylan backbone recovered from both AS-CTMP pretreated materials.

Acetate commonly esterifies the O-3 and to a lesser extent the O-2 hydroxyls of the xylan backbone. In contrast, ferulate can occur as phenolic-free structures esterifying the O-5 hydroxyls of arabinosyl side groups attached to the xylan backbones. Diferulate structures cross-linking two xylan backbones can also occur to a minor extent. Another possibility for ferulate in grass cell walls is that it bridges the original phenolic-free hydroxyls to lignin, forming 4-O-ether structures in LCCs.^38^ Therefore, the occurrence of significant levels of 4-O-ether-linked ferulate structures in samples extracted from BM substrates suggests that the endoxylanase-assisted extraction procedure released LCCs in addition to xylan fractions. In contrast, the presence of the same structures in the extracts from alkaline-sulfite pretreated materials (Table 1) suggests that these 4-O-ether-linked ferulates are associated with the extracted lignin fractions but present acid-free structures at the Cγ of the propyl side chain.^39^

Phenolic-free ferulates were of special interest in the current work because extracted xylans containing these side groups are reactive in cross-linking reactions initiated by laccases.^40,41^ Phenolic-free *p*-coumarate could also react with laccases but it has been widely accepted that *p*-coumarate radicals quickly undergo radical transfer to other phenolic structures, which limits their oligomerization or direct cross-link with lignin.^42,43^ The amount of phenolic groups in the samples is another indicator of the sample’s suitability to react with laccase. These phenolic groups can originate from phenolic-free ferulate besides from phenolic-free *p*-coumarate and phenolic groups occurring in lignin and LCCs. The total amount of phenolic groups in the evaluated samples was 0.25 ± 0.01% and 0.35 ± 0.01% for BM and AS-CTMP sugarcane bagasse, and 0.27 ± 0.02% and 0.46 ± 0.01% for BM and AS-CTMP corn stover, respectively, indicating that all samples have available reaction sites for oxidation by laccase. In contrast, the oat spelt reference xylan contained only 0.020 ± 0.001% of phenolic structures, which is a value close to the detection limit of the analytical method.^27^

**Figure 1:**
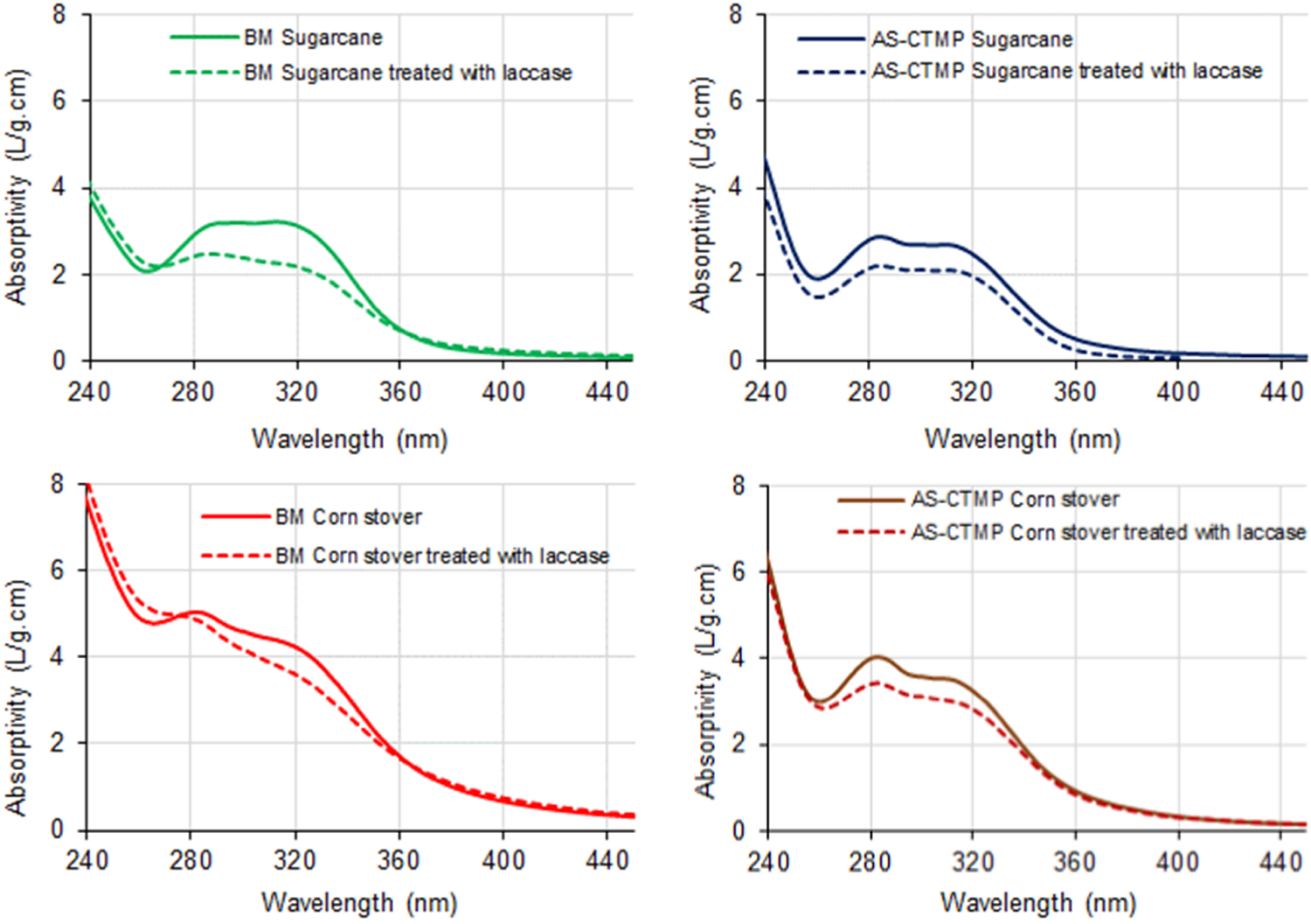
UV/vis spectra of water-soluble products recovered after digestion of ball-milled (BM) and alkaline-sulfite chemithermomechanically pretreated (AS-CTMP) sugarcane bagasse and corn stover with 8 IU/g substrate of alkali-active endoxylanase. Untreated samples are represented by continuous lines, while treated samples, shown with dashed lines, correspond to each extract treated with laccase at 200 IU/g of substrate for 2 h at 30 °C.

**Figure 2:**
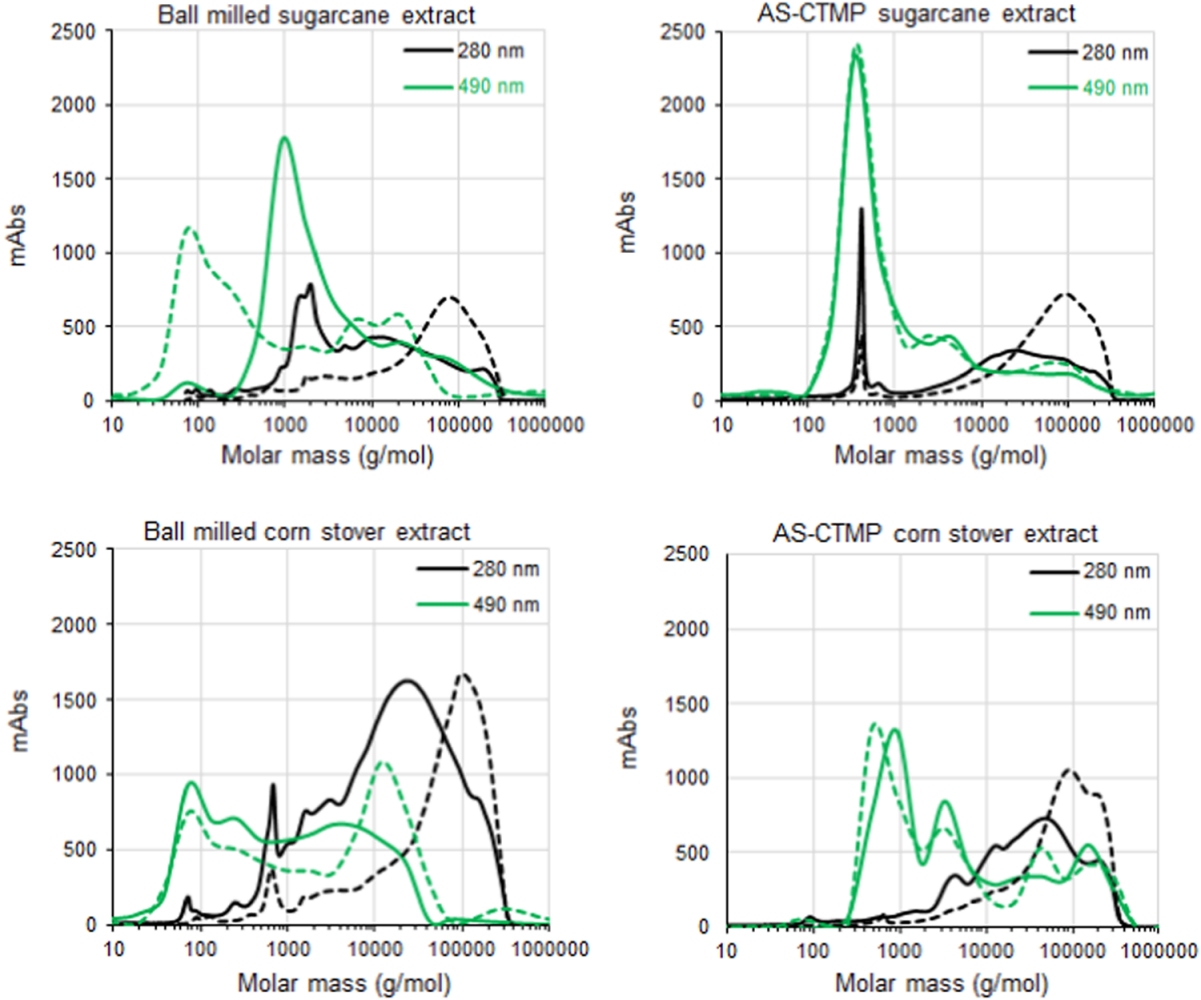
Size exclusion chromatography of water-soluble products recovered after digestion of ball-milled and alkaline-sulfite chemithermomechanically pretreated (AS-CTMP) sugarcane bagasse and corn stover with 8 IU/g substrate of alkali-active endoxylanase. Untreated samples are represented by continuous lines, while treated samples, shown with dashed lines, correspond to each extract treated with laccase at 200 IU/g substrate for 2 h at 30 °C.

The mixtures of xylans and LCCs were subsequently treated with *T. versicolor* laccase, and the reaction products were analyzed by using UV/vis spectroscopy and size exclusion chromatography (SEC) (Figures 1 and 2, respectively).

UV/vis spectra of treated samples showed a typical decrease in the 315 nm absorptivity, suggesting the opening of the α-β unsaturation of the *p*-hydroxycinnamates contained in the samples.^34^ Eluted SEC fractions were detected online for aromatic compounds at 280 nm, while carbohydrate fractions were identified through a postcolumn reaction with the phenol-sulfuric acid reagent at 490 nm.^1^ General SEC analysis revealed that the untreated control samples from sugarcane bagasse and corn stover exhibited polydisperse characteristics, confirming that the extracts contained a diverse mixture of oligosaccharides, LCCs, and water-soluble lignin fractions. After laccase treatment, the aromatic fractions from all samples exhibited a significant shift in molar mass distribution toward higher values (Figure 2). In several samples, this polymerized aromatic fraction eluted on the exclusion limit of the column, which was 70 kDa. In contrast, the carbohydrate fractions exhibited different behaviors, primarily influenced by the type of pretreatment. The carbohydrate fractions from AS-CTMP samples were largely unaffected by laccase treatment, whereas those from the BM substrates separated into two distinct fractions. One of the carbohydrate fractions showed a significant shift toward higher molar masses, while the other consisted of monomers or short oligomeric sugars, likely formed by the hydrolysis of the glucan portion of these samples (Table 1). This is attributed to the presence of detectable levels of endoglucanases and β-glucosidases in the laccase extract.^23^ Collectively, SEC data suggest that extracts from BM substrates contained a carbohydrate fraction enriched with free-phenolic ferulates, facilitating cross-linking after laccase treatment, whereas the AS-CTMP substrates primarily reacted through the phenolic components of their water-soluble lignin fractions.

### Laccase-Mediated Reactions Providing Xylans and LCCs Incorporation into High-Yield Kraft Fibers

The laccase-mediated oxidation of the water-soluble products was investigated to facilitate the incorporation of xylan and LCCs into the HYEKP fibers. Previous studies have focused on the incorporation of xylan into bleachable-grade pulp fibers, where nearly pure xylans are required to minimize the need for excessive bleaching agents in subsequent steps.^44,45^ In such cases, xylan adsorbed onto pulp fibers increases the pulp yield and improves pulp quality. However, the primary drawback of this approach is the low retention of xylan in the pulp after bleaching.^17,44,45^

In the current work, the hypothesis was that crude extracts containing xylan and LCC mixtures could be covalently fastened to the lignin in HYEKP fibers, resulting in modified fibers with increased wettability – a limiting characteristic of currently available brown tissue paper furnish. This type of pulp furnish could help in preparing more sustainable tissue paper because it can be obtained at high yield and avoids harmful bleaching steps.^12,14,15^

**Figure 3:**
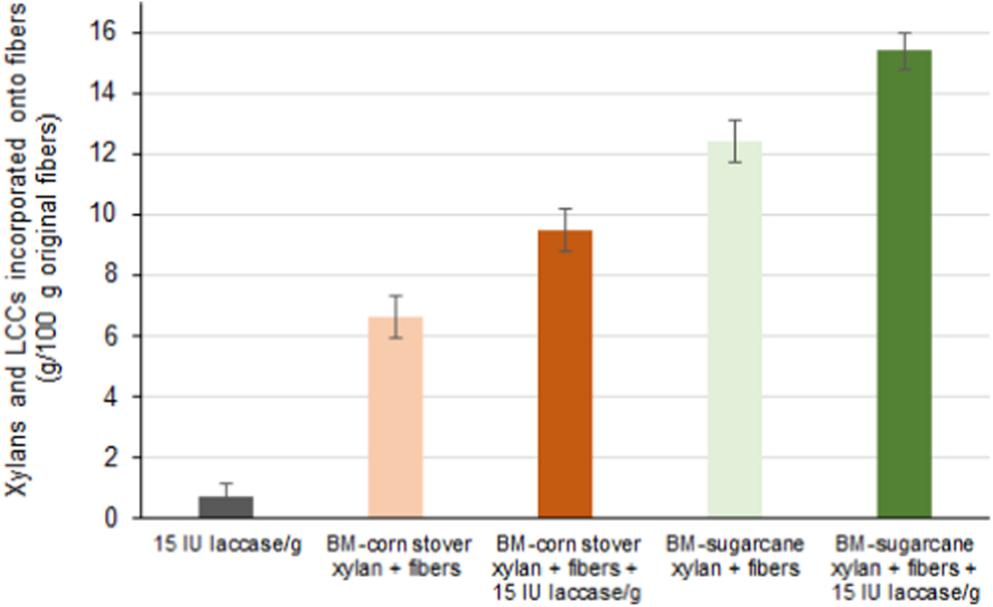
Incorporation of xylans and lignin-carbohydrate complexes from ball-milled (BM) pretreated samples into high-yield eucalyptus kraft pulp fibers in laccase-mediated reactions. Incorporation levels of a reference xylan from oat spelt, as well as the extracts recovered from alkaline-sulfite chemithermomechanically pretreated materials, were below 1% (w/w) and considered the threshold for the current experiments.

HYEKP fibers were obtained at 68% (w/w) pulp yield and contained 19.0 ± 0.9% (w/w) total lignin as well as 59.7 ± 1.1% (w/w) glucan, 12.2 ± 0.2% (w/w) hemicellulose, and 1.3 ± 0.1% (w/w) ash. The incorporation of xylans and LCCs into these fibers was evaluated by measuring the mass increase in the pulps after treatment (Figure 3). Xylan from oat spelt served as a reference sample due to its lack of phenolic groups (0.02% w/w). In this case, as well as in the cases of the extracts recovered from AS-CTMP pretreated materials, the mass of treated fibers increased less than 1% (w/w). This level of xylan incorporation into bleachable-grade pulp fibers (with lignin contents below 2%) has already been reported and attributed to xylans that remain adsorbed on the fibers through strong hydrogen bonding and/or occlusion in fiber pores.^17,44,45^ In contrast, the HYEKP fibers treated with extracts recovered from BM pretreatment showed a significantly higher mass increase, ranging from 6.6 ± 0.7% (w/w) to 12.4 ± 0.7% (w/w) (Figure 3). The extracts prepared from BM materials contained acetylated and feruloylated xylans and LCCs, which could interact with both polysaccharides and lignin in the HYEKP fibers. The use of laccase-mediated reactions further increased the incorporation of xylans and LCCs, reaching 9.5 ± 0.7% (w/w) for BM corn stover extracts and 15.4 ± 0.6% (w/w) for sugarcane bagasse extracts. Interestingly, the extracts recovered from BM-pretreated materials underwent intense polymerization of the carbohydrate fraction after laccase treatment (Figure 2), suggesting that their phenolic-free *p*-hydroxycinnamates were key components promoting cross-linking with the lignin in the pulp fibers.

**Figure 4:**
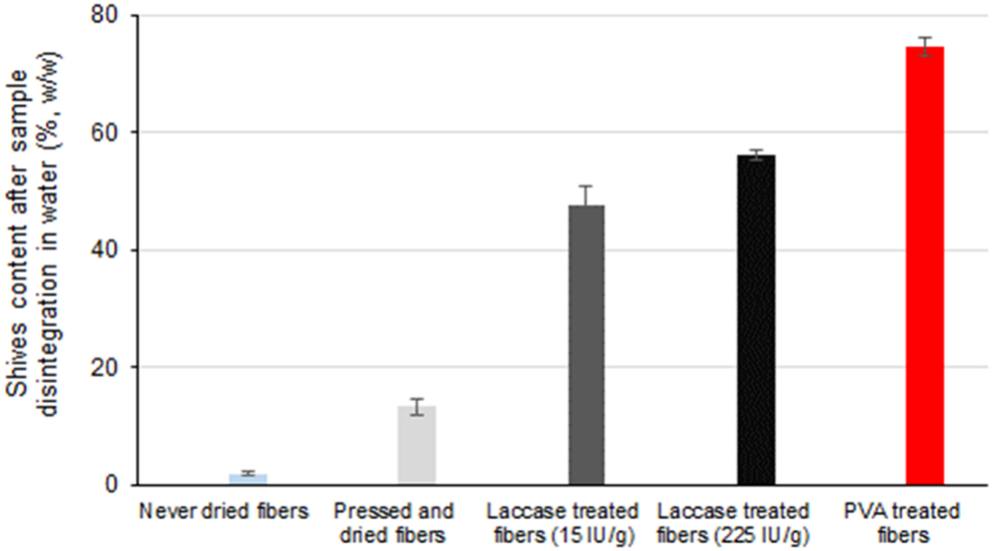
Fiber shives detected in high-yield eucalyptus kraft pulp after the disintegration of test specimens in a water suspension. Never-dried fibers served as the untreated control, while pressed and dried fibers were used as the reference sample. A water-soluble adhesive (poly(vinyl alcohol)) was used as the positive control in the assay.

**Figure 5:**
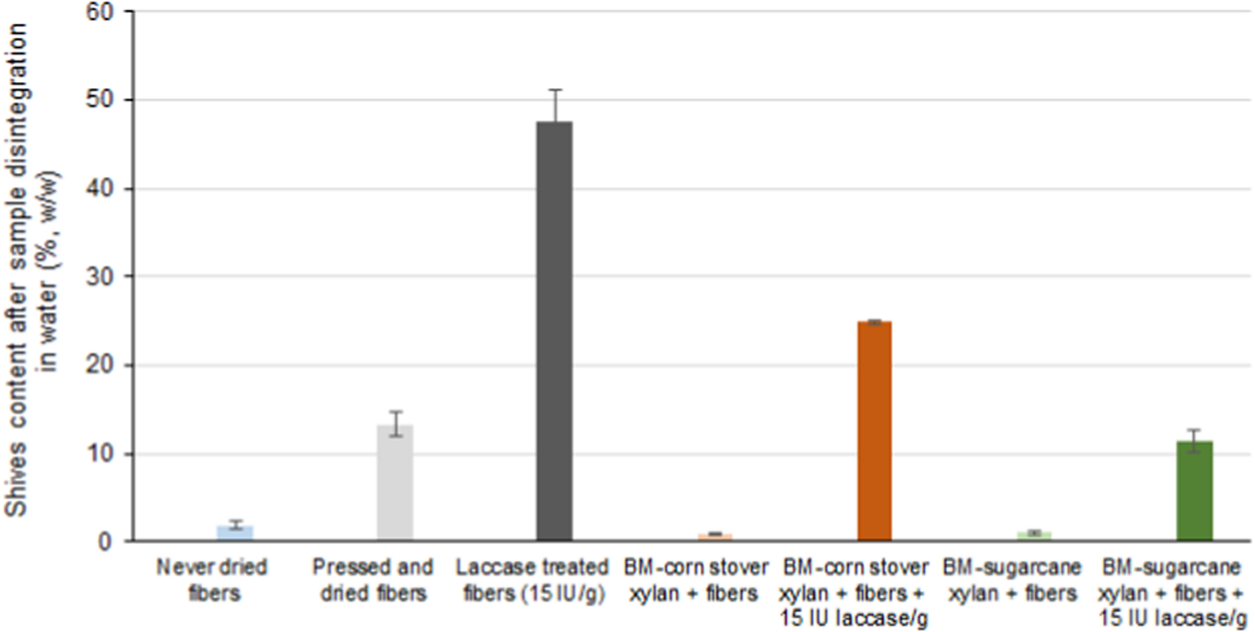
Effect of incorporated xylans and lignin-carbohydrate complexes on high-yield kraft pulp fibers was assessed by measuring the fiber shives detected after the disintegration of the test specimens in a water suspension. Never-dried fibers served as the untreated control, while pressed and dried fibers served as the reference sample.

Laccase treatment of mechanical pulp fibers used in medium-density fiberboard production has long been reported to increase fiber–fiber interactions, including the cross-linking of lignin on fiber surfaces.^20^ Additionally, lignin-containing fibers oxidized by NaIO_4_ treatment have also provided strong fiber–fiber interactions in the production of medium-density fiberboard, medium-density particleboard, and plywood, without the use of harmful formaldehyde-based adhesives.^46^ Similarly, the current laccase-mediated reactions applied to HYEKP fibers could potentially enhance the paper strength characteristics. To evaluate fiber–fiber interactions after laccase treatment of HYEKP fibers, we further disintegrated the pressed and dried fibers in water using a standard procedure (TAPPI T205). The amount of fiber shives resisting the pulp disintegration procedure was an indication of strong fiber–fiber interactions (Figure 4). As expected, never-dried HYEKP fibers easily disintegrated in a water suspension, producing a very low number of fiber shives. In contrast, a water-soluble adhesive (poly(vinyl alcohol)), used as a positive control, produced a very high amount of fiber shives, resisting disintegration in the water suspension (74.7 ± 1.6% w/w), thereby confirming the reliability of the currently evaluated method for indicating strong fiber–fiber interactions. The laccase treatment of HYEKP fibers increased the shives content in a dose-dependent manner, corroborating strong fiber–fiber interactions after enzyme-mediated reactions.^20^ When xylans and LCCs were incorporated onto the fibers without laccase treatment, the shives content after pulp disintegration decreased significantly, most likely because the adsorbed xylans and LCCs prevented strong fiber–fiber interactions (Figure 5). The action of the laccase on the same samples partially restored the fiber–fiber interactions, resulting in shive contents similar to those observed in fibers treated with laccase alone, without xylans and LCCs.

**Figure 6:**
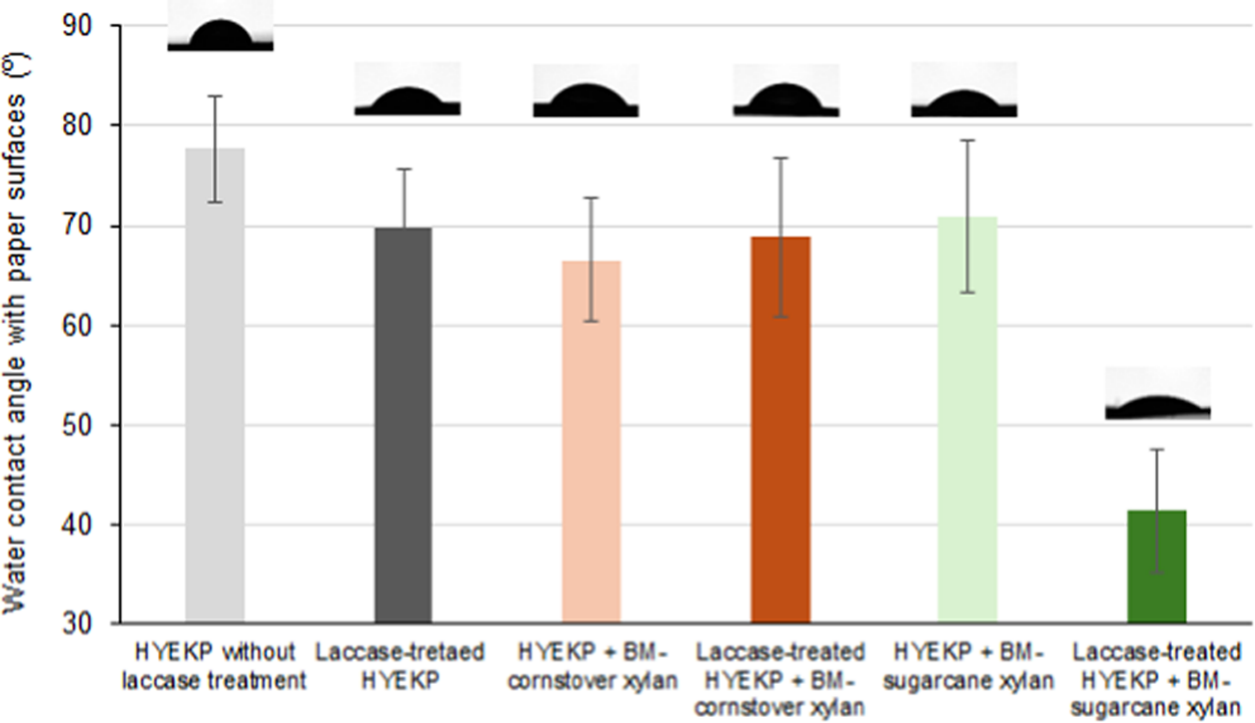
Water contact angle on paper sheet surfaces prepared from high-yield eucalyptus kraft pulp (HYEKP) fibers with and without incorporation of xylans and lignin-carbohydrate complexes recovered from ball-milled (BM) corn stover or sugarcane bagasse. Bars represent the average water contact angles and corresponding standard deviations (*n* = 3), while the images illustrate drops on the paper sheet surfaces after 0.5 s.

Considering the potential use of HYEKP fibers for the production of brown tissue paper furnish, the wettability of paper sheet surfaces made from these fibers is a key characteristic.^12^ The water contact angle of paper sheets prepared from the currently evaluated samples was measured to assess their wettability, as more wettable paper surfaces absorb water quickly, resulting in very low water contact angles.^31^ The fully bleached reference pulp sample produced highly wettable paper sheet surfaces, resulting in low water contact angles that, when detectable, ranged from 30° to 40°, corroborating previous studies on bleached kraft pulps.^16,31^ In the case of the HYEKP fibers, the average water contact angle was 77 ± 5°, reflecting its relatively high-lignin content (19.0 ± 0.9% w/w) (Figure 6). The water contact angle decreased in all treated samples but particularly in the sample with the highest level of xylans and LCCs incorporation (15.4% w/w for xylans/LCCs from BM sugarcane bagasse treated with laccase). In this case, the water contact angle of the treated HYEKP (41 ± 6°) reached values similar to those detected for fully bleached eucalyptus kraft pulps.

The incorporation of xylans and LCCs into the fibers was further evaluated by using X-ray photoelectron spectroscopy (XPS) on paper sheets prepared from treated fibers that incorporated high amounts of xylans and LCCs. This incorporation can occur through adsorption processes, which depend on the chemical structure of the fractions as well as their molar mass distribution,^17,44,45^ or through cross-linking resulting from the laccase-mediated reactions of phenolic structures.^19^ Low-resolution XPS data provided information about the O/C ratio of the paper surfaces (Table 3). The O/C ratio detected in the acetone-extracted reference filter paper was 0.75, which is close to the theoretical value for pure cellulose (0.83). In the HYEKP, this value decreased to 0.57, likely due to the presence of 19.0 ± 0.9% (w/w) lignin, which has an average O/C ratio of approximately 0.33.^30^ The incorporation of corn stover or sugarcane bagasse xylans and LCCs into these fibers did not significantly change the O/C ratios. Theoretical O/C ratios for the HYEKP fibers and the water-soluble products were calculated based on original values observed in lignin and polysaccharides as well as the chemical composition of each material (Table 1). Weighing the chemical composition of the incorporated extracts (Table 1) and their incorporation levels (Figure 3) indicates that the O/C ratios would indeed change only within the range of standard deviations of the low-resolution XPS measurements.

**Table 3:** O/C Ratios Estimated from Low-Resolution XPS of Paper Surfaces Prepared from High-Yield Eucalyptus Kraft Pulp Fibers with and without Incorporation of Xylans and Lignin-Carbohydrate Complexes (LCCs) Derived from Ball-Milled (BM) Sugarcane Bagasse and Corn Stover

sample	O/C
fiber	0.57 ± 0.02
fiber + laccase	0.54 ± 0.01
fiber + BM corn stover xylan/LCC	0.58 ± 0.01
fiber + BM corn stover xylan/LCC + laccase	0.52 ± 0.01
fiber + sugarcane xylan/LCC	0.56 ± 0.01
fiber + sugarcane xylan/LCC + laccase	0.52 ± 0.01
extracted filter paper (reference sample)	0.75 ± 0.02

The high-resolution XPS data provided relevant information, particularly based on the O–C=O (C4) signal (Table 4). While C1–C3 and C5 did not show significant changes among samples, the C4 contents were diagnostic of xylans and LCCs incorporation onto the fibers, as only ester and acid groups present in xylans and LCCs carry these functional groups (acetyl, ferulate, and 4-*O*-methyl glucuronic acids in xylans and *p*-coumarate in LCCs and grass lignins). This signal increased in samples incorporating xylan and LCCs after laccase treatment, with the highest difference observed for crude fibers (0.48 ± 0.14) compared to Fiber + Sugarcane xylan/LCC + Laccase (0.86 ± 0.05).

**Table 4:** High-Resolution XPS Data Recorded for Paper Surfaces Prepared from High-Yield Eucalyptus Kraft Pulp Fibers with and without Xylan and Lignin-Carbohydrate Complexes (LCCs) Incorporation in Laccase-Mediated Reactions

	homogeneous equivalent composition, *x*/10^–2^				
sample	C1	C2	C3	C4	C5
fiber	13.69 ± 1.44	41.05 ± 0.92	7.1 ± 0.39	0.48 ± 0.14	0.73 ± 0.06
fiber + laccase	16.64 ± 1.10	39.57 ± 0.60	6.84 ± 0.19	0.57 ± 0.17	0.87 ± 0.16
fiber + BM corn stover xylan/LCC	12.19 ± 0.20	41.94 ± 0.26	7.43 ± 0.04	0.52 ± 0.02	0.73 ± 0.07
fiber + BM corn stover xylan/LCC + laccase	12.68 ± 0.51	41.3 ± 0.21	7.73 ± 0.13	0.65 ± 0.08	0.61 ± 0.19
fiber + sugarcane xylan/LCC	13.33 ± 0.34	41.57 ± 0.21	7.33 ± 0.13	0.65 ± 0.07	0.73 ± 0.15
fiber + sugarcane xylan/LCC + laccase	16.81 ± 0.99	40.23 ± 0.63	6.27 ± 0.31	0.86 ± 0.05	1.21 ± 0.17
extracted filter paper (reference sample)	2.70 ± 0.16	43.21 ± 0.34	10.02 ± 0.17	0.94 ± 0.30	

## Conclusions

Feruloylated xylans and lignin-carbohydrate complexes (LCCs) extracted from ball-milled corn stover and sugarcane bagasse were successfully incorporated into HYEKP, with incorporation levels reaching up to 15.7 g per 100 g of fibers. The highest incorporation levels of xylans and LCCs were achieved through laccase-mediated reactions, likely due to cross-linking between the phenolic structures of both materials. High-resolution XPS analysis of the paper surfaces confirmed the incorporation of xylans, as evidenced by an increase in the intensity of the O−C=O (C4) signal, which is associated with acetyl and ferulate groups, in the samples with higher xylan and LCC levels. The incorporated xylans and LCCs reduced the water contact angle on the paper surfaces. These findings suggest that the addition of these components could enhance not only the yield but also the wettability of brown tissue paper furnish.

## Supporting Information Available

Illustration of the formation of test specimens used to evaluate xylan and LCC incorporation into high-yield eucalyptus kraft pulp fibers, the size distribution of ball-milled pretreated biomass, UV/vis spectra of water-soluble products extracted via the xylanase-assisted procedure, and the chemical composition of pretreated solids (PDF)
