# Supplementary material for: Laccase-Mediated Incorporation of Xylans and Lignin-Carbohydrate Complexes into High-Yield Eucalyptus Kraft Fibers
Source: ACS Omega. 2025 Apr 17;10(16):16863–73. doi: 10.1021/acsomega.5c00812 (PMC12044572; doi:10.1021/acsomega.5c00812)
Supplement: Supplementary file 1 — ao5c00812_si_001.pdf [file ao5c00812_si_001.pdf]

Laccase-mediated incorporation of xylans and lignin-carbohydrate complexes into high-yield eucalyptus kraft fibers

Uirajá Cayowa Magalhães Ruschoni[1], Pedro Jorge Fonseca Chagas[1], Pieter De Wever[2], Samuel Eyley[3], Wim Thielemans[3], Adriane Maria Ferreira Milagres[1], Pedro Fardim[2], André Ferraz[1*]

1. Departamento de Biotecnologia, Escola de Engenharia de Lorena, Universidade de São Paulo, 12602-810 Lorena, SP, Brazil.

2. Department of Chemical Engineering, KU Leuven, Celestijnenlaan 200F, 3001 Leuven, Belgium

3. Sustainable Materials Laboratory, Department of Chemical Engineering, KU Leuven, Kulak Kortrijk Campus, Etienne Sabbelaan 53, 8500 Kortrijk, Belgium

(*) andreferraz@usp.br

Supporting information

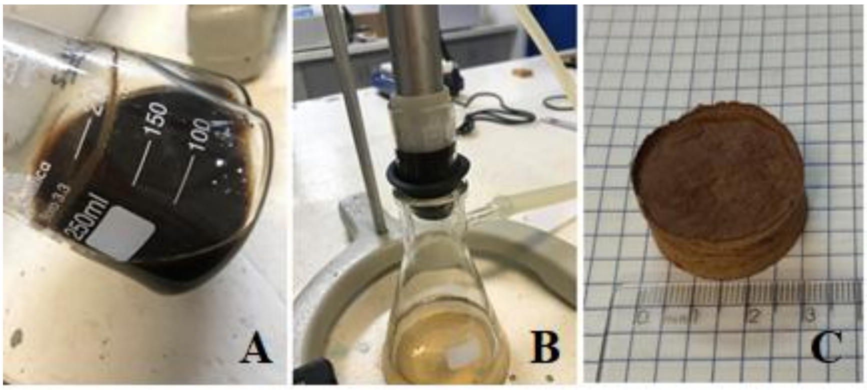

Figure S1. Formation of test specimens used in the evaluation of xylans and LCC incorporation into high-yield eucalyptus kraft pulp fibers. Fiber suspensions were mixed with xylans and LCCs extracts and let to react in laccase-mediated reactions (**A**). The treated fiber suspensions were vacuum filtered through sintered glass filters (Schott #3) and washed with 200 mL of water. After washing, the fibers were pressed with a 1-kg stainless steel solid billet under vacuum for 1 min (**B**). Pressed fibers were dried at 60 ºC for 16h and then at 100 ºC for additional 4h and weighed (**C**).

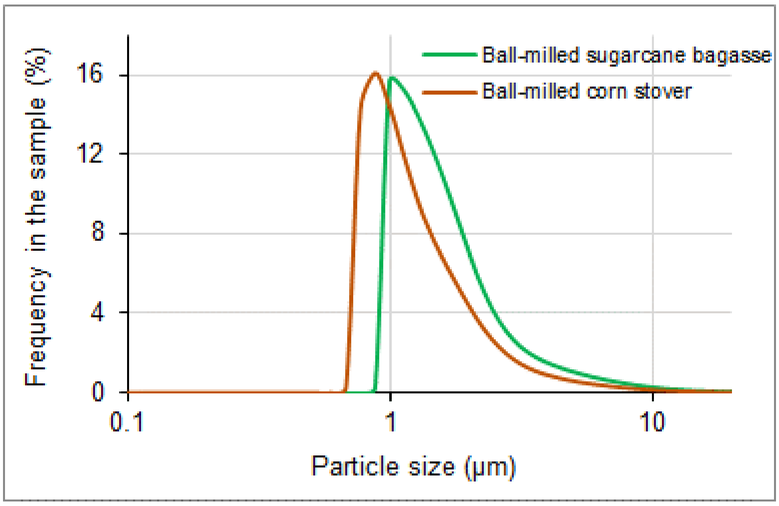

Figure S2. Size distribution of sugarcane bagasse and corn stover samples after ball milling pretreatment. Lignocellulosic materials were milled to flour appearance in a 3.7 L stainless steel jar set in the ball-milling equipment (Quimis Q298, Brazil). Each 90 g of extractive-free material was crushed by 1.8 kg of 18-mm stainless balls for 130 h at 98 rpm.

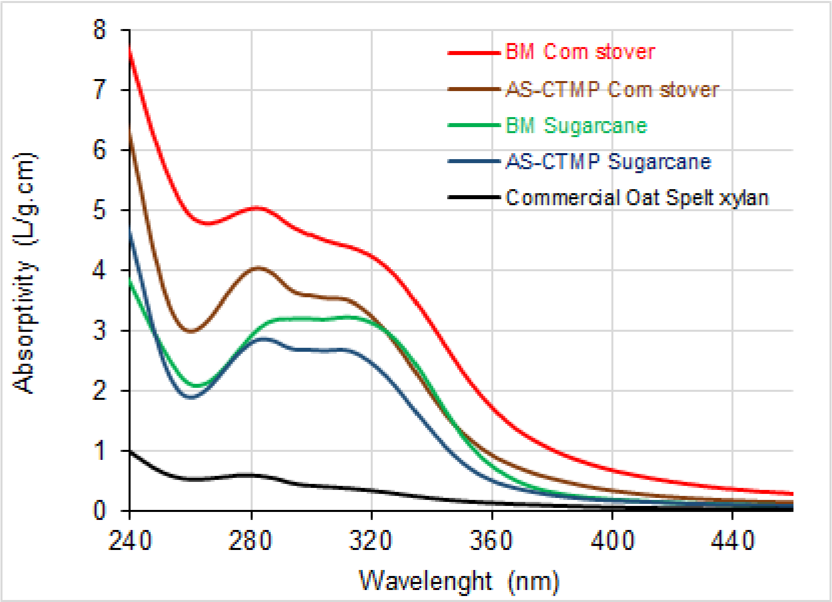

Figure S3. UV/Vis spectra of water-soluble products recovered after digestion of ball-milled (BM) and alkaline-sulfite chemithermomechanically pretreated (AS-CTMP) sugarcane bagasse and corn stover with 8 IU/g substrate of alkali-active endoxylanase. Absorptivity values were calculated from the ash- and glucan-free concentrations of each sample.

Table S1. Yield of pretreated solids, chemical composition and mass balance for plant biomass components after ball-milling (BM) and alkaline-sulfite chemithermomechanical (AS-CTMP) pretreatments.

Biomass sample	Yield of pretreated solids (%)	Chemical composition of biomass sample (g/100 g biomass sample)					Mass balance of bagasse components (g /100 g of original sugarcane bagasse)				
		Lignin	Glucan	Xylan	Arabinosyl	Acetyl	Lignin	Glucan	Xylan	Arabinosyl	Acetyl
*Original sugarcane bagasse**											
	100	20.3±1.2	40.2±0.6	21.6±0.3	2.1±0.1	3.2±0.1	20.3	40.2	21.6	2.1	3.2
Pretreated sugarcane bagasse											
BM**	97.3	20.9±1.2	41.4±0.6	22.2±0.6	2.2±0.1	3.3±0.1	20.3	40.2	21.6	2.1	3.2
AS-CTMP***	80.1	12.6±0.2	45.7±0.1	22.9±0.1	2.5±0.1	0.3±0.1	9.9	36.6	18.3	2.0	0.2
*Original corn stover**											
	100	20.2±0.2	31.3±1.1	16.3±0.7	2.0±0.1	2.3±0.1	20.2	31.3	16.3	2.0	2.3
Pretreated corn stover											
BM**	84.9	23.8±0.2	36.9±1.1	19.2±0.7	2.3±0.1	2.7±0.1	20.2	31.3	16.3	2.0	2.3
AS-CTMP***	66.4	17.5±0.2	46.0±0.1	21.1±0.1	2.8±0.1	1.1±0.5	11.6	30.5	14.0	1.9	0.7

(*) Ethanol-soluble extractives and ash contents in sugarcane bagasse were 2.7 ± 0.1 and 3.8 ± 0.3 g/100 g, and 15.1 and 5.8 g/100 g in corn stover, respectively.
(**) Prior to ball-milling pretreatment, ethanol-soluble components were quantitatively extracted from plant biomass materials. Therefore, reported yields of the pretreated solids results from extractives removal in the process.
(***) Each alkaline-sulfite pretreatment was performed with 1.2 kg plant biomass and 12 L alkaline-sulfite white liquor containing 3.75% NaOH and 7.5% Na_2SO_3 (g/100 g of sugarcane bagasse) at 127 °C for 2 h. Cooked solids were subsequently refined with 32 L water in a disk refiner. The yield of pretreated solids after AS-CTMP reflected mainly lignin and acetate removal (Heinz et al., 2022); however, in the case of corn stover, the low yields of pretreated solids also reflected extensive removal of ethanol-soluble components that were significantly high in the original untreated material.
